# Assessment of mandibular bony healing, mandibular condyle and angulus after orthognathic surgery using fractal dimension method

**DOI:** 10.4317/medoral.26587

**Published:** 2024-04-14

**Authors:** Ozge Muftuoglu, Hakan Alpay Karasu

**Affiliations:** 1 Orcid:0000-0001-7993-2406. Assistant Professor, Department of Orthodontics, Faculty of Dentistry, Ankara Medipol University, Ankara, Turkey; 2 Orcid:0000-0003-1867-0347. Professor, Department of Oral and Maxillofacial Surgery, Faculty of Dentistry, Ankara Medipol University, Ankara, Turkey

## Abstract

**Background:**

This study aims to compare the trabeculation changes in the bone structure observed at the mandibular osteotomy line and the mandibular condyle in patients after single and double-jaw orthognathic surgery.

**Material and Methods:**

The study included 38 patients (23 female, 15 male) who underwent mandibular surgery with bilateral sagittal split ramus osteotomy technique. The patients were divided into two groups according to their surgical operation: single-jaw (bilateral sagittal split ramus osteotomy) or double-jaw (Le Fort I osteotomy and bilateral sagittal split ramus osteotomy) surgery. Trabecular changes seen in mandibular osteotomy lines, mandibular condyle and mandibular angulus were evaluated on panoramic radiographs of patients (preoperative, postoperative 2nd day, postoperative 3rd month and 12th month) using fractal analysis method. Fractal dimension analysis was calculated by box counting method.

**Results:**

No statistically significant difference was found between the fractal analysis values in the mandibular condyle and angulus region preoperatively, postoperative 2nd day, postoperative 3rd month and postoperative 12th month in the single jaw group. There was no statistically significant difference between the fractal analysis values in the mandibular condyle and angulus region preoperatively, postoperative 2nd day, postoperative 3rd month and postoperative 12th month in the double jaw group. A significant difference was found in fractal analysis values in osteotomy lines in both groups. The lowest value was found on the 2nd postoperative day and reached the preoperative values in the 3rd and 12th months postoperatively. Fractal analysis values didn't show significant difference between the single, double-jaw groups in all periods.

**Conclusions:**

This result suggests that the fractal analysis method can be used to evaluate trabeculation in the bone healing process of the osteotomy lines and indirectly affected areas in the postoperative period after orthognathic surgery.

** Key words:**Bone regeneration, fractal analysis, mandible, orthognathic surgery.

## Introduction

Bone healing is a regenerative process involving many complex cellular activities. In a short time, the inflammatory response is triggered in the healing area and the bone tissue returns to its original physical and mechanical properties with the secreted growth factors. Healing occurs in three stages: the early inflammation stage, the repair stage, and the late remodeling stage (1).

Fracture healing is completed in the remodeling stage, where the healed bone returns to its original shape, structure and mechanical strength. Bone remodeling happens gradually over months and years (2).

Determining when a fracture has healed is crucial for returning to function, the appropriate time to remove screws and plates, and diagnosing clinical conditions such as nonunion. There is no consensus on the determination of fracture healing. Detection of fracture healing is essential for surgeons dealing with trauma. Still, the lack of consensus on this issue can lead to controversial results and expose patients to avoidable risks (3).

Due to its low cost and easy accessibility, radiographs are frequently used to evaluate fracture healing. The formation, development of the callus and the bridging of the fracture lines with the callus can be observed in the radiographs. Dental panoramic radiographs are routinely used to detect fractures in traumas in the maxillofacial region. Bone density can be evaluated on panoramic radiographs (4-6).

Maxillofacial deformities have adverse effects on the appearance and daily life of individuals. These deformities not only cause defects in facial appearance and occlusion but also affect the psychosocial state of individuals. Le Fort I osteotomy and bilateral sagittal split ramus osteotomy (BSSO) are widely used to correct maxillofacial deformities (7-10).

Fractal analysis (FA) is a mathematical method in which body structures can be evaluated, and the result of this method is defined as a fractal dimension (FD). Fractal analysis is an accurate, economical and effective method for evaluating bone tissue and is used safely in medicine and dentistry. In panoramic radiographs, the FD calculation is used to evaluate the mineral density, structure of trabecular bone and to identify bone changes (11-13).

Following up on bone healing in patients after orthognathic surgery is essential. This study aims to evaluate bone changes at the osteotomy line, angulus and mandibular condyle using FA in patients who had single and double-jaw surgery on panoramic radiographs.

## Material and Methods

This retrospective study was approved by the local ethics committee of our institution (Protocol No: 62). This study was carried out in line with the Helsinki Declaration of 1975, as revised in 2000. Consent was obtained from the patients.

Inclusion criterias:

1. Patients with Class III skeletal anomaly and treated with orthognathic surgery

2. No prior history of orthodontic/orthognathic surgery

3. Patients with high-quality panoramic radiographs, before surgery (T0), 2 days after surgery (T1), 3 months after surgery (T2), and 1 year after surgery (T3)

4. Fixation with bicortical screw in BSSO

5. Patients who recovered without complications

Exclusion criterias:

1. Patients with the syndrome

2. Patients who use drugs that impair bone metabolism or have a disease

Two groups were formed in the study, single-jaw and double-jaw surgery. BSSO was performed to the mandible in the single-jaw group. Le Fort I osteotomy was performed to the maxilla and BSSO to the mandible in the double-jaw group.

38 patients (23 female, 15 male) were included in this study. The age range of the patients was between 18 and 26, with a mean of 21±2,34.

- Panoramic Radiography Protocol:

All panoramic radiographs were obtained using a single device (Castellini X-Radius Tr10 Plus, Italy) with settings of 60-85 kVp, 4-8mA, and an exposure time of 12.3 seconds. The radiographs of all patients were taken by the same radiology technician and positioned by the manufacturer's recommendations. Frankfurt horizontal plane was regulated parallel to the ground, and the sagittal plane was leveled with the vertical line. To ensure intra-examiner reliability and calibration of the evaluations, the same observer reviewed the images again two weeks after the initial assessment.

**Figure 1:**
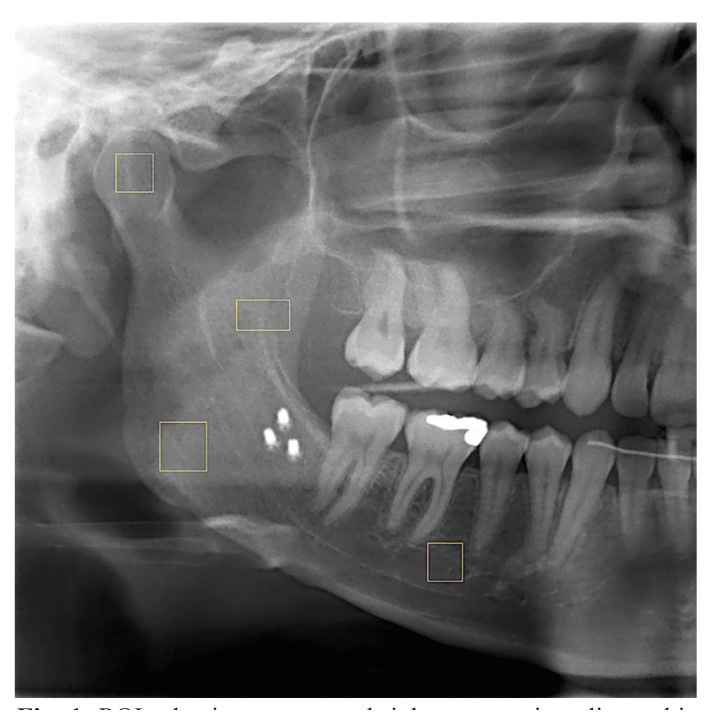
ROI selection on cropped right panoramic radiographic images.

FD analysis was calculated by box counting method using ImageJ (14) (National Institutes of Health, Bethesda, MD, USA) program on it. For the standardization, the right mandible was examined in the study. Four regions of interest (ROI) were selected from panoramic radiography. ROI-1 was designed as a 45 x 45 pixel square at the geometric center of the mandibular condyle’s superior. ROI-2 was determined as the geometric center of the mandibular angulus, a square of 60 × 60 pixels. ROI-3 was determined as the vertical osteotomy line on the corpus, as a 45 x 45 pixel square. ROI-4 was determined as the horizontal osteotomy line on the ramus, superior to the lingula of mandible, a rectangle placed of 90 × 40 pixels (Fig. 1).

**Figure 2:**
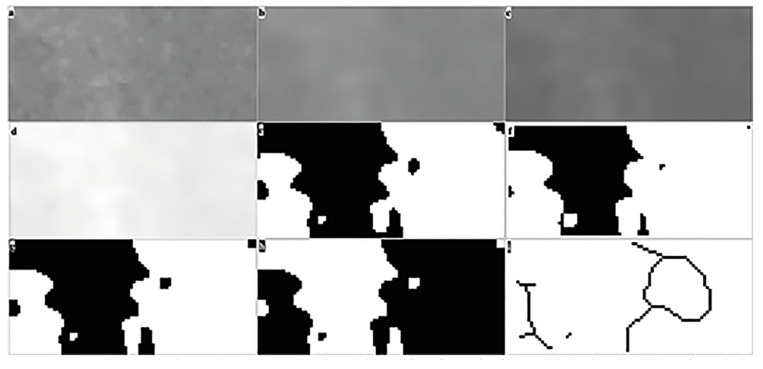
Fractal analysis methods: a. Selected ROI and duplicated, b. Addition of Gaussian filter, c. Subtraction, d. Addition of 128 pixels, e. Binarized version, f. Eroded version, g. Dilated version, h. Inverted version, i. Skeletonization.

The image was duplicated after selecting the ROI (Fig. 2). To achieve a blurred effect, a Gaussian filter was applied to the image (Fig. 2). The resulting blurred image was then subtracted from the original image (Fig. 2). To enlarge certain features with varying brightness, such as trabeculae and bone marrow, 128 gray values were added to each pixel location, generating a new image (Fig. 2). By setting a brightness value of 128 as a threshold, the image was converted into a binary image (Fig. 2). To reduce noise, the binary image underwent erosion and dilation processes (Fig. 2). The image was then inverted, representing trabeculae as black and the bone marrow as white (Fig. 2). Ultimately, the image underwent a skeletonization process, progressively removing pixels until only a central pixel line remained (Fig. 2). The software utilized a box counting algorithm, dividing the image into squares of 2, 3, 4, 6, 8, 12, 16, 32, and 64 pixels (Fig. 2). The number of frames containing trabeculae and the total number of frames were calculated for each pixel size. The obtained values were plotted on a logarithmic scale graph. The slope of the line drawn through the plotted points on the graph provided the FD value.

- Statistical Analysis

IBM SPSS Statistics version 21.0 (IBM Corp., Armonk NY, USA) was used to analyze the data. Descriptive statistics were performed for minimum-maximum values, mean and standard deviations. The Shapiro-Wilk test was used to evaluate whether the distribution was normal. The t-test was used to compare the single or double-jaw groups. In comparing the period values, if the distribution was normal, the repeated ANOVA test and the post hoc Tukey test were used. If the distribution was not normal, the Friedman test and the post hoc Dunn test were used. The statistical significance level was accepted at *p*<0.05.

## Results

A total of 38 patients consisted in the study, including 22 double jaw and 16 single jaw surgery. The mean age was 20.46 ± 1.97 years in the double-jaw group and 22.08 ± 2.61 years in the single-jaw group. Demographic data are presented in Table 1.

The intraclass correlation coefficient calculated as 0.917 (*p*<0.001). The ROI values of the mandibular condyle, angulus and osteotomy lines according to the periods in patients with single-jaw and double-jaw are given in Table 2.

A statistically significant difference was found between periods in ROI-3 in double-jaw patients (*p*<0.001). When periods were compared with each other, significant difference was found between the preoperative and the 2nd day (*p*<0.001), between the 2nd day and the 3rd month (*p*<0.001) and between the 2nd day and the 12th month (*p*<0.001).

A statistically significant difference was found in ROI-4 compared to periods in double jaw patients (*p*<0.001). When periods were compared with each other, between preoperative and 2nd day (*p*<0.001), between preoperative and 3rd month (*p*=0.023), between 2nd day and 3rd month (*p*=0.002) and between 2nd day and 12th month statistically significant difference was found (*p*<0.001).

A statistically significant difference was found between periods in ROI-3 in single-jaw surgery patients (*p*=0.006). When periods were compared with each other, significant difference was found between the pre-op and the 2nd day (*p*=0.021), between the 2nd day and the 3rd month (*p*=0.024) and between the 2nd day and the 12th month (*p*=0.009).

A statistically significant difference was found in ROI-4 when compared between periods in single-jaw surgery patients (*p*= 0.038). When periods were compared, only significant difference was found between the pre-op and the 2nd day (*p*=0.032).

The changes of the FA values between the groups in the time intervals were shown in Table 3. There was no statistically significant difference between FA values in single and double-jaw surgery patients at all periods.

## Discussion

FA is widely used in dentistry and successfully evaluates bone structures on panoramic radiographs (15). Although cone beam computed tomography (CBCT) is considered the gold standard in dentistry today, it has disadvantages, such as high radiation dose and cost compared to panoramic radiographs. Panoramic radiographs are used safely in planning before bone surgeries in dentistry, such as dental implant surgery (16). For this reason, we carried out our study by using panoramic radiography images taken routinely for preoperative and postoperative evaluation in orthognathic surgery patients.

The sagittal split ramus osteotomy is the most commonly used osteotomy to correct mandibular deformities. Bicortical screws or mini plates and monocortical screws are most commonly used for fixation. Fixation with 3 bicortical screws placed on the ascending ramus has shown good stability and no stability difference between fixation with mini-plate and monocortical screws (17,18). In this study, we included patients fixed with bicortical screws to prevent artifacts in the vertical osteotomy area and obtain a reliable FA value.

Bone healing is essential for returning to normal life after orthognathic surgery. Heo *et al*. (12) evaluated osteotomy areas after mandibular orthognathic surgery with FA at various periods. They reported a statistically significant difference between the periods; the lowest value was observed on the 1st and 2nd days after the procedure, and the FA value increased over time.

Podcernina *et al*. (19) evaluated the structural changes in the condyle and the volume of the condyle on CBCT in patients with single and double-jaw surgery patients. They reported no difference in condylar volume and structural changes in both groups at all periods. Similarly, no statistically significant difference was found in bone trabeculation of the mandibular condyle at all periods in the single and double-jaw surgery groups in our study.

In their study, Colak *et al*. (20) evaluated vertical and horizontal osteotomies on 30 patients with BSSO and reported that FA values decreased in the early postoperative period and increased in the postoperative 6th and 12th months. Similar to these studies, the FA value decreased significantly in the early postoperative period after single and double-jaw surgery in our study. It increased in the postoperative 3rd and 12th months, and there was no significant difference between the preoperative values in the 12th month in both groups.

Coban *et al*. (21) evaluated various regions in patients who underwent BSSO with genioplasty and only genioplasty. In the angulus region, they didn't find a significant difference in FA values in the two groups, preoperatively and 6 months after surgery. Similar to this study, no significant difference was found in FA values in the angulus region at all periods in both groups in this study.

When the single and double-jaw surgery groups were compared in our study, no statistically significant difference was found in the changes in FA values in all regions and all periods.

To our knowledge, no study in the literature compares bone healing in single and double-jaw surgery with FA. Our study shows no difference in the healing of the mandible as a result of single and double-jaw surgeries performed after good planning.

The limitation of our study is that FA values were not evaluated on CBCT. Future studies with more different techniques and more patients are required.

## Conclusions

According to the results of this study, BSSO osteotomy lines showed the lowest FA values on postoperative day 2 in both groups. FA values tended to return to their initial values in the postoperative period. Based on these data, clinicians can recommend when patients can return to their daily routines. The fractal analysis method can be considered reliable and effective in evaluating bone healing after BSSO with panoramic radiographs.

## Figures and Tables

**Table 1:** Demographic data of the study groups.

Study group	Female	Male	Total	Age (Mean ± SD)
Double Jaw	14	8	22	20.46±1.97
Single Jaw	9	7	16	22.08± 2.61

**Table 2:** Fractal dimensions of each period according to region of interest.

Period	ROI-1		ROI-2		ROI-3		ROI-4	
	SingleJaw	DoubleJaw	SingleJaw	DoubleJaw	SingleJaw	DoubleJaw	SingleJaw	DoubleJaw
Preoperative	0.6±0.09	1.06±0.09	1.14±0.13	1.09±0.09	1.06±0.11	1.05±0.08	1.12±0.13	1.1±0.13
Postoperative 2nd Day	1.04±0.09	1.04±0.09	1.13±0.13	1.08±0.09	0.9±0.09	0.89±0.07	0.89±0.14	0.94±0.11
Postoperative 3rd Month	1.07±0.07	1.04±0.1	1.09±0.13	1.11±0.08	1.06±0.09	1.03±0.11	1.02±0.12	1.03±0.11
Postoperative 12th Month	1.04±0.09	1.06±0.09	1.12±0.12	1.13±0.11	1.08±0.1	1.07±0.09	1.08±008	1.04±0.1
*p*	0.942	0.582	0.917	0.421	0.006*	<0.001*	0.038*	<0.001*

* *p* < .05

**Table 3:** Comparison of fractal dimension differences between single and double-jaw groups in the time intervals.

Regions of interest (ROI) and Study group		T0-T1		T1-T2		T2-T3	
		(Mean±SD)	*p*	(Mean±SD)	*p*	(Mean±SD)	*p*
ROI-1	Single-Jaw	0.0212±0.0020	0.999	-0.0292±0.146	0.275	0.0280±0.109	0.208
	Double-Jaw	0.0212±0.0018		0.0053±0.107		-0.0231±0.109	
ROI-2	Single-Jaw	0.0114±0.0013	0.575	0.0288±0.159	0.377	-0.0193±0.115	0.845
	Double-Jaw	0.0109±0.001		-0.0249±0.121		-0.0180±0.0977	
ROI-3	Single-Jaw	0.159±0.0167	0.768	-0.155±0.125	0.377	-0.020±0.146	0.247
	Double-Jaw	0.157±0.0115		-0.175±0.0982		0.0341±0.0949	
ROI-4	Single-Jaw	0.169±0.0196	0.728	-0.0608±0.215	0.642	-0.0590±0.0983	0.303
	Double-Jaw	0.165±0.0194		-0.0930±0.128		-0.0115±0.0981	
